# Candidates for Intra-Articular Administration Therapeutics and Therapies of Osteoarthritis

**DOI:** 10.3390/ijms22073594

**Published:** 2021-03-30

**Authors:** Eriko Toyoda, Miki Maehara, Masahiko Watanabe, Masato Sato

**Affiliations:** 1 Department of Orthopaedic Surgery, Surgical Science, Tokai University School of Medicine, 143 Shimokasuya, Isehara 259-1193, Japan; etoyoda@tokai-u.jp (E.T.); m-maehara@tsc.u-tokai.ac.jp (M.M.); masahiko@is.icc.u-tokai.ac.jp (M.W.); 2 Center for Musculoskeletal Innovative Research and Advancement (C-MiRA), Tokai University Graduate School of Medicine, 143 Shimokasuya, Isehara 259-1193, Japan

**Keywords:** disease-modifying osteoarthritis drug, gene therapy, oligonucleotide therapeutics

## Abstract

Osteoarthritis (OA) of the knee is a disease that significantly decreases the quality of life due to joint deformation and pain caused by degeneration of articular cartilage. Since the degeneration of cartilage is irreversible, intervention from an early stage and control throughout life is important for OA treatment. For the treatment of early OA, the development of a disease-modifying osteoarthritis drug (DMOAD) for intra-articular (IA) injection, which is attracting attention as a point-of-care therapy, is desired. In recent years, the molecular mechanisms involved in OA progression have been clarified while new types of drug development methods based on gene sequences have been established. In addition to conventional chemical compounds and protein therapeutics, the development of DMOAD from the new modalities such as gene therapy and oligonucleotide therapeutics is accelerating. In this review, we have summarized the current status and challenges of DMOAD for IA injection, especially for protein therapeutics, gene therapy, and oligonucleotide therapeutics.

## 1. Introduction

Osteoarthritis (OA) of the knee is a disease in which degeneration of articular cartilage gradually progresses, and the cartilage is eventually worn to expose the subchondral bone. OA significantly decreases the quality of life due to joint deformation and pain. While most patients with OA are elderly, it has been reported that nearly 40% of people over the age of 60 years have signs of early OA [1,2]. In addition to aging, OA is a disease involving multiple etiologies such as a history of joint injury, gender, genetic background, and obesity [3,4]. Since degenerated cartilage does not repair spontaneously, it is important to prevent the degeneration of cartilage from an early stage. However, current drug treatment for early OA is limited to the administration of analgesics for pain, viscosupplement such as hyaluronic acid that enhances joint lubricity, and intra-articular (IA) injection of corticosteroids [5]. None of these therapies are considered to have the effect of suppressing the progression of cartilage degeneration and destruction and ultimately, replacement arthroplasty is the only radical treatment for late-stage OA. Since OA is not a life-threatening disease, minimally invasive treatment at an outpatient level is preferred; IA injection is most commonly employed for OA. Since OA progresses over a long period of time, early intervention is important so that symptoms can be controlled throughout life. The development of a disease-modifying osteoarthritis drug (DMOAD) that can be administered by IA injection, which is attracting attention as a point-of-care therapeutic, is desired.

Currently, corticosteroids and hyaluronic acid are the drugs most commonly administered by IA injection, with the main action being pain control. So far, no DMOADs have been developed [6]. Novel treatment options are being developed with the expectation of controlling inflammation and promoting cartilage regeneration in OA. Options include recombinant protein, gene therapy, platelet-rich plasma, and cell therapy [7]. This review outlines the current status and challenges that the new biologics bring to treating OA, especially protein therapeutics, gene therapy, and nucleic-acid therapeutics.

## 2. Target Biological Pathways for DMOADs

OA is a disease mainly characterized by cartilage degeneration, pathological changes in entire joint, such as joint capsule thickening, osteophyte formation, subchondral sclerosis, and synovitis. Although the molecular mechanism underlying these pathological conditions remains not fully understood, many excellent reviews that discuss the molecular pathology of OA have been published [6,8,9,10,11,12]. As a consensus, prolonged low-grade inflammation is thought to play a pivotal role in OA initiation and progression. In summary, activation of innate immunity by the decomposition products of the joint matrix generated by trauma or mechanical overload causes synovitis. Synovitis induces production of proinflammatory mediators from synovial cells, immune cells, chondrocytes, or cells in subchondral bone. Risk factors for OA, such as aging, injury history, obesity, and some genetic backgrounds are thought to trigger or prolong inflammation.

Increased proinflammatory cytokines such as interleukin (IL)1β, tumor necrosis factor α (TNF-α), IL-6, IL-15, IL-17, and IL-18, and the imbalance of anti-inflammatory cytokines such as transforming growth factor-β (TGFβ), IL-4, IL-10, and IL-13 suggested contribute to OA pathogenesis [13,14,15].

Further, inflammatory joint condition induces alteration of the chondrocyte phenotype, such as cell proliferation, cluster formation, production of both matrix proteins and matrix-degrading enzymes, and chondrocyte hypertrophy and apoptosis [16]. Matrix-degrading enzymes such as matrix metalloproteinases (MMPs) and a disintegrin and metalloproteinase with thrombospondin motifs (ADAMTS) family degrade cartilage component type II collagen and proteoglycan. Alteration of chondrocyte phenotype considered as regenerative response caused by inflammation. The chondrocytes follow the process of chondrocyte maturation and resulting matrix remodeling, improper hypertrophy-like maturation and cartilage calcification. Cartilage homeostasis disruption is considered to be involved in cartilage degeneration and osteophyte formation.

The importance of each factor in OA progression remains unclear. A blockage of the inflammatory signal induced by proinflammatory mediators, a supplementation of anti-inflammatory factors, or an alteration of chondrocyte phenotype is considered a promising target for DMOADs.

## 3. The Advantage of IA Delivery in OA Treatment

For the control of inflammation, protein therapeutics blocking TNFα, IL-1, and IL-6 have been already developed for treatment of rheumatoid arthritis (RA). RA is a systemic inflammatory disease and also causes joint destruction. Inflammation of RA is generally more severe than that of OA. Thus, the control of inflammation by these protein therapeutics is of benefit to the RA patients and increases in the occurrence of infections is tolerated [17,18].

In contrast to RA, the daily symptoms in OA such as morning stiffness, heat, pain, and joint effusions are initially comparatively mild [8]. The systemic administration of immunosuppressive protein therapeutics poses an unacceptable risk for OA patients.

In OA, usually only one or two large joints are affected, and inflammation is limited to the affected joints. Furthermore, articular cartilage, which is the target tissue for OA treatment, is an avascular tissue, which makes it difficult for a drug to be distributed by systemic administration. IA drug delivery is relatively easy for the diarthrodial joint, thereby ensuring that the drug is delivered to the affected area. The merit of IA injection is that sufficient drug concentration can be achieved at the target site while avoiding the disadvantages of potential side effects. Thus, IA delivery of DMOADs, including already developed immunosuppressive protein therapeutics, has been considered as an option to address the issues, adverse effect, and drug distribution.

## 4. OA Treatment by IA Injection of Protein Therapeutics

As mentioned above, various inflammatory cytokines and cartilage matrix-degrading enzymes in joint fluid increase. The expression of genes encoding these proteins in synovial cells and chondrocytes also increases to the extent that suppression of TNFα and IL-1β actions is considered to be a promising target for the treatment of OA. In addition, IA injection of humoral factors that have cartilage repair and cartilage protection properties is also of great interest because they may be a DMOAD for OA of the knee (Table 1).

IL-1Ra, which is a natural antagonist of IL-1β, and anti-IL-1β antibody, which neutralizes IL-1β, have already been shown to be effective in RA. Clinical trials targeting OA have also been conducted. Considering the potent induction of matrix-degrading enzymes by IL-1β, IL-1Ra may suppress the degradation of articular cartilage. However, in a clinical trial (NCT00110916) of IA injection of IL-1Ra (anakinra) [19], the drug failed to achieve the primary endpoint, a change in the Western Ontario and McMaster Universities osteoarthritis index (WOMAC) score from baseline to week 4 [20]. It has been pointed out that there is the possibility the cartilage protective effect had not been exhibited because of the short half-life of IL-1Ra (4 h) and because patient-relevant pain assessment data were used as the primary endpoint [21]. In future studies, it is considered necessary to maintain the IA concentration of IL-1Ra at an effective concentration by improving the dwell time, such as with a controlled release of the recombinant protein. Currently, a clinical trial of anakinra aimed at preventing OA by administration within 7 days after anterior cruciate ligament (ACL) injury is scheduled (NCT03968913 [22]). Since it is estimated that 50% of patients with ACL injury develop OA, the trial plan is to track the progression to OA by measuring marker proteins in joint fluid for early inflammation and cartilage damage. The effect of IL-1Ra could be observed even with a temporary effect.

**Table 1:** Clinical trials of intra-articular (IA) injection of recombinant protein for osteoarthritis (OA) patients.

Study Name (ClinicalTrials.gov ^1^)	Mode of Action Biologicals	Study Phase Outcome Measures	Study Identifier Current Status ^1^ (Completion Year)
Biologic Therapy to Prevent Osteoarthritis After ACL Injury	Inhibit IL-1β IL-1 Receptor antagonist r-metHuIL-1ra (Anakinra)	Early Phase 1 cytokine level/Knee pain and function/marker level	NCT03968913 [22] Not yet recruiting
Study of Safety, Tolerability, Preliminary Efficacy of Intra-articular LNA043 Injections in Patients with Articular Cartilage Lesions and Knee Osteoarthritis	Assist cartilage repair. A modified human angiopoietin-like 3 protein	Phase 2 MRI/AEs/protein level/antibodies/Others	NCT03275064 [23] 2017~Recruiting
A Study to Investigate the Safety and Effectiveness of Different Doses of Sprifermin in Participants with Osteoarthritis of the Knee (FORWARD)	Assist cartilage repair Fibroblast growth factor 18 (Sprifermin)	Phase 2 MRI/WOMAC/PGA/mJSW/Protein level	NCT01919164 [24] Completed (2019) has results
Dose Finding Study of Bone Morphogenetic Protein 7 (BMP-7) in Subjects with Osteoarthritis (OA) of the Knee	Assist cartilage repair Bone Morphogenetic Protein 7 (BMP-7/OP-1)	Phase 2 WOMAC	NCT01111045 [25] Completed (2011)
To Determine the Safety, Tolerability, Pharmacokinetics and Effect on Pain of a Single Intra-articular Administration of Canakinumab in Patients with Osteoarthritis in the Knee	Inhibit IL-1β humanized monoclonal antibody to interleukin-1β (Canakinumab)	Phase 2 AEs/VAS/WOMAC/Others	NCT01160822 [26] Completed (2010) has results
Treatment of Knee Osteoarthritis with Intra-Articular Infliximab	Inhibit TNFα chimeric monoclonal antibody to TNF-α (Infliximab)	Phase 4 Cellular infiltrates/Effusion/WOMAC/Others	NCT01144143 [27] Completed (2011) has results
Study of Intra-articular DLX105 Applied to Patients with Severely Painful Osteoarthritis of the Knee	Inhibit TNFα a single-chain (scFv) antibody fragment against TNF-α (DLX105)	Phase 1/2 AEs/VAS/WOMAC	NCT00819572 [28] Completed (2010)

^1^https://clinicaltrials.gov (accessed on January 2021), the most recent phase and status study of the same drug candidate. ACL, anterior cruciate ligament; WOMAC, Western Ontario and McMaster osteoarthritis index (WOMAC) pain subscale; MRI, quantification or assessment of cartilage by MRI; AEs, incidence of adverse events; PGA, Patient’s Global Assessment; mJSW, Minimal Joint Space Width in the Medial and Lateral Compartments as Evaluated by X-ray; VAS, Visual Analogue Scale to assess the intensity of knee pain.

The IL-1β neutralizing antibody, canakinumab, is effective against juvenile idiopathic arthritis [29]. A clinical trial of IA injection of canakinumab in OA patients was conducted in 2012 (NCT01160822 [26]). Further study of this drug for the treatment of OA has not been conducted. Interestingly, in the Canakinumab Anti-inflammatory Thrombosis Outcomes Study, a clinical trial in atherosclerosis patients involving 10,061 participants, patients received drug subcutaneously once every 3 months. Study results report that inhibition of IL-1β with canakinumab may substantially reduce rates of total hip arthroplasty/total knee replacement and ameliorate osteoarthritis-related symptoms [30]. Although verification is necessary, these results suggested that persistent inhibition of IL-1β might suppress OA progression. If subcutaneous administration of canakinumab is safe and tolerated, it may be an option for DMOAD.

Clinical trials have been conducted to determine the efficacy of a TNFα neutralizing antibody (infliximab) and a single-chain (scFv) antibody fragment against TNF-α, both of which inhibit the action of TNFα. For infliximab, the suppression of synovial inflammation had not been observed. For antibody fragment, no results have been posted and subsequent studies have not been performed.

An alternative to suppression of inflammation as an approach to DMOADs, IA injection of protein factors that promote chondrocyte differentiation and inhibit cartilage hypertrophy, has been carried out in anticipation of cartilage protection and promotion of regeneration. Human recombinant bone morphogenetic protein/osteogenic protein-1 (BMP-7/OP-1) is a factor that has been reported to suppress chondrocyte hypertrophy and protect cartilage. It had been used to promote bone regeneration [31]. Clinical trials of BMP-7 in OA showed that in participants who received BMP-7, there was a trend toward greater symptomatic improvement than with a placebo [25,32]; however, rhBMP-7 have been withdrawn from the market because of lingering safety concerns; vertebral osteolysis, ectopic bone formation, radiculitis, or cervical soft tissue swelling [33], and further study for OA treatment have not been conducted.

FGF18 has been confirmed as both inducing cartilage differentiation and having a protective action [34,35]. A clinical trial of IA injection of rhFGF18 (Sprifermin) was conducted, but no clear improvement was observed in the pain evaluation [24,36,37]. However, a dose-dependent increase in cartilage thickness has been observed, and there are high expectations for its potential as a DMOAD [38,39,40].

Angiopoietin-like 3 (ANGPTL3) has been identified as an inducer of chondrogenesis and cartilage repair. Clinical trials of IA injection of a modified form of ANGPTL3 are under way [41].

Provided that articular cartilage repair is expected, chondrocytes that respond to these factors or progenitor cells capable of cartilage differentiation need to be present or supplied to the degenerated cartilage. Chondroprogenitor cells have been reported to be present in synovial cells, cartilage surface cells, and periosteal cells, and are expected to contribute to joint protection [42,43]. Encouraging results with rhFGF18 [38,39,40] imply that the ability to respond to regenerative stimuli may remain in the joints of OA patients.

The development of protein therapeutics has been accelerated and many other proteins have been shown to be effective in animal OA models (Table 2). Protein engineering is increasingly used aiming at enhancing the efficacy and improving biodistribution and bioavailability.

## 5. Gene Therapy for OA Treatment by IA

In recent years, gene therapy with viral vectors containing plasmid deoxy ribonucleic acid (DNA) or messenger ribonucleic acid (mRNA) that incorporates genes with therapeutic effects has been put into practical use. Gene therapy exerts its effects through intracellular nucleic-acid transfection and translation into protein. In the case of gene therapy with mRNA, it does not need to be transcribed intracellularly, protein can be produced directly from the mRNA. When aiming for continuous expression, mRNA transfection is considered to be inferior to gene transfer by vectors. Since mRNA is of very low stability, drug delivery system (DDS) techniques that incorporate mRNA into cells while avoiding degradation are necessary. Lipid nanoparticles (LNPs) are currently the mainstream DDS for mRNA transfection but they are difficult to target to particular cell types, other than hepatocytes, because most LNPs are trapped by the liver when administered systemically. IA injection of the LNP-encapsulated mRNA may maintain protein bioavailability and action on synovial cells or chondrocytes in the target joint before the LNPs circulate to the whole body. Gene therapy with mRNA administration could be suitable for transient gene expression aimed at controlling inflammation after trauma and suppressing chondrocyte apoptosis.

Currently, research is being conducted on therapeutic methods in which vectors and plasmids are administered to cells in joints to produce factors with anti-inflammatory and cartilage repair effects instead of IA administration of recombinant proteins. Since OA is not a life-threatening disease, a vector with a higher safety profile than those used for cancer treatment is required. Table 3 shows the clinical studies of IA gene therapy for OA conducted to date. The viral vectors currently in clinical trials were adeno-associated virus (AAV) vectors, helper-dependent adenovirus (HDAd) vectors, and plasmids.

AAV is a nonpathogenic virus that can be transfected into cells in a tissue-specific manner, depending on their serotype, and can transfer genes into nondividing cells. Their use is expanding due to those characteristics. The introduction of AAV vectors into joints has been investigated in rat [57], rabbit [58], and equine [59] species, and is introduced into chondrocytes. Since adult chondrocytes usually do not proliferate and are rarely replaced, it is expected that long-term transgene expression will be possible, if genes can be introduced into chondrocytes [60]. If anti-inflammatory proteins can be continuously supplied through gene therapy, it may be possible to modify OA progression. In this context, gene therapy utilizing an IL-1Ra gene-containing vector has major implications for OA treatment based on the results obtained with protein administration. Viral transfection of the potent anti-inflammatory cytokines, IL-10, and IFNβ is also being investigated.

**Table 3:** Clinical trials of intra-articular delivery of gene therapy for OA patients.

Study Name (ClinicalTrials.gov ^1^)	Mode of Action Biologicals	Study Phase Outcome Measures	Study Identifier Current Status ^1^ (Completion Year)
Safety of Intra-Articular Sc-rAAV2.5IL-1Ra in Subjects with Moderate Knee OA (AAVIL-1Ra)	Inhibit IL-1β sc-rAAV2.5IL-1Ra	Phase 1 AEs	NCT02790723 [61] 2019~Recruiting
Study to Evaluate the Safety and Tolerability of FX201 in Patients with Osteoarthritis of the Knee	Inhibit IL-1β humantakinogene hadenovec IL-1Ra (FX201)	Phase 1 AEs/biodistribution	NCT04119687 [62] 2020~Recruiting
Efficacy and Safety of XT-150 in Osteoarthritis of the Knee	Supply IL-10 plasmid DNA with a variant of human IL-10 transgene (XT-150)	Phase 2 KOOS/WOMAC /Others	NCT04124042 [63] 2020~Recruiting
A Single Dose Clinical Trial to Study the Safety of ART-I02 in Patients with Arthritis	Supply IFN-β Recombinant AAV type 2/5 containing a hIFN-b gene (ART-I02)	Phase 1 AEs/clinical scores distribution/immune response /Others	NCT02727764 [64] Active (2022)

^1^https://clinicaltrials.gov (accessed on January 2021), the most recent phase and status study of the investigational drug. AEs, incidence of adverse events; KOOS, Osteoarthritis Outcome Score; WOMAC, Western Ontario and McMaster Osteoarthritis Index pain subscale.

A clinical trial of protein therapeutics of IL-1Ra showed that IL-1Ra rapidly disappeared from the joint. However, clinical trials of FX201 (humantakinogene hadenovec) aiming to express IL-1Ra in the joint are under way (NCT04119687 [62]). FX201 is an HDAd vector based on human serotype 5 (Ad5) that is designed to express IL-1Ra under the control of an inflammation-sensitive promoter. HDAd is a vector that lacks all viral genes except the regions required for viral genome replication and packaging to increase the safety of adenovirus vectors. It is a nonintegrating, nonreplicating vector that can incorporate relatively long genes. IA expression of IL-1Ra using HDAd in mice and rats has been reported [65]. It has also been reported that cotransfection with the HDAd vector containing the gene coding for lubricin exhibited improved protection against OA than single IL-1Ra coding HDAd vector transfection [66].

Similarly, aiming for IA expression of IL-1Ra, sc-rAAV2.5rIL-1Ra (a self-complementing, recombinant adeno-associated viral vector carrying IL-1Ra complementary DNA) has been investigated in a clinical trial (NCT02790723 [61]). The conventional AAV vector is single-stranded, and gene expression occurs after the complementary strand is synthesized in the cell to become double-stranded. In the process of self-complementing, the positive and negative strands are connected and packaged into a viral capsid to quickly become double-stranded, and hence gene expression occurs rapidly [67]. It is inferior to conventional AAV in that large-sized genes cannot be introduced. Preclinical studies have confirmed that more than 90% of the post-IA vector in rats remains in the joint and that IL-1Ra levels are also elevated [68]. Furthermore, improvement in total joint pathology following gene therapy has been reported in an equine OA model [69].

IL-10 is a potent anti-inflammatory cytokine and is thought to affect OA. IL-10 suppresses the production of IL-1β, IL-6, and TNF-α and induces the expression of IL-1Ra [70]. In addition, it suppresses matrix metallopeptidases (MMPs) expression. IL-10 is expected to be able to broadly suppress the inflammatory response and cartilage destruction seen in OA [71]. A plasmid DNA-based therapy (XT-50) that expresses a long-acting human IL-10 variant has been developed. The variant (hIL-10var) is inserted under the control of a C-X-C motif chemokine ligand 10 promoter and the promoter is expected to regulate IL-10 expression according to the onset of inflammation in the joint [72]. Administration of XT-150 to canine joints has reportedly increased IA IL-10 levels and reduced pain [73]. Clinical studies of a plasmid vector (XT-150) on OA have been conducted (NCT04124042 [59]).

IFNβ also regulates and suppresses the production of TNFα, IL-1β, and IL-6, and an AAV vector (ART-I02) that expresses IFNβ is being studied in rheumatoid arthritis and OA. ART-I02 is designed to express IFNβ under the control of a nuclear factor κB (NF-κB) promoter, which acts as a transcription factor during the induction of many inflammatory cytokines. More than 90% of the virus stayed in the joint after IA administration of ART-I02 and protein expression was confirmed even after 7 weeks [74]. In the administration of ART-I02 for collagen-induced arthritis in rhesus monkeys, a beneficial effect on joint swelling, histological inflammation, and bone erosion scores has been reported [75]. ART-I02 is also undergoing clinical research targeting OA (NCT02727764 [60]).

Introduction of PRG4/lubricin, which plays an important role in cartilage integrity, was investigated. Using 10mabHDV-PRG4, HDAd vector conjugated to an α-10 integrin monoclonal antibody, the increase of transduction into chondrocytes and production of lubricin have been reported [76]. Introduction of *IGF-I* by AAV vector has been also investigated [77]. Since the protein can be continuously expressed, gene therapy considered one of the options for improving the biodistribution and bioavailability. Using gene therapy, it is possible in principle to express a range of receptors and transcription factors, and thus, it may be possible to use gene therapy to change cellular properties such as the differentiation potential of chondrocytes. The safety and usefulness of gene therapy in OA treatment is yet unknown. It is expected that the knowledge of the production of neutralizing antibodies and the efficiency of introduction into target cells by IA in human bodies will be clarified from the ongoing clinical trials in the future.

## 6. Oligonucleotide Therapeutics as a Candidate for OA Treatment by IA Injection

In recent years, the development of oligonucleotide therapeutics that introduce oligo nucleic acids such as antisense and small interfering RNA (siRNA) into cells has progressed. The major oligonucleotide therapeutics are double-stranded RNA, siRNA [78], micro RNA (miRNA) [79], and single-strand RNA antisense [80]. Antisense is classified into splicing-controlled antisense that interferes with pre-mRNA splicing [81], RNA-degrading antisense that collaborates with RNaseH to degrade mRNA, and miRNA-inhibiting antisense that inhibits miRNA function [80]. In the past, in vivo administration of nucleic acid has had problems of intracellular delivery and in vivo stability. Over the past decade, modified nucleic acids have been developed that have improved nuclease resistance and increased stability in the body. By chemically modifying the nucleic acid, the binding property to the target sequence and the efficiency of uptake into cells have also been improved [82,83,84,85]. So far, antisense and siRNA have been put into clinical use [86,87,88,89,90,91,92,93,94,95].

For OA treatment, attempts are being made to suppress cartilage destruction by controlling the production of protein molecules involved in pathological conditions such as cytokines and signal transduction molecules using oligonucleotide therapeutics. In recent years, many comprehensive analyses of miRNAs and mRNAs in OA joints have been carried out, and the networks of mRNAs and miRNAs involved in the progression of OA have been clarified [96,97,98]. Many miRNAs involved in OA and chondrogenesis have been reported [99,100,101]. For genes involved in promoting OA, there is the possibility that OA progression could be suppressed by degrading specific mRNAs with siRNA or antisense RNA and suppressing protein production. In the case where miRNAs that target proteins associated with cartilage destruction in OA are reduced, administration of oligonucleotide therapeutics may be able to supplement the anabolic miRNAs and suppress OA progression. Conversely, using an antisense RNA to inhibit miRNAs that are increased in OA, it may be possible to restore cartilage protective protein production, which is suppressed by these miRNAs and control OA progression.

Table 4 shows oligonucleotides that exhibited in vivo efficacy in experimental OA models, those are limited in rodent models. Application of oligonucleotide therapeutics for IA injection is still underway. So far, no oligonucleotide therapeutics for DMOADs is in clinical trials yet.

## 7. Discussion

**Figure 1:**
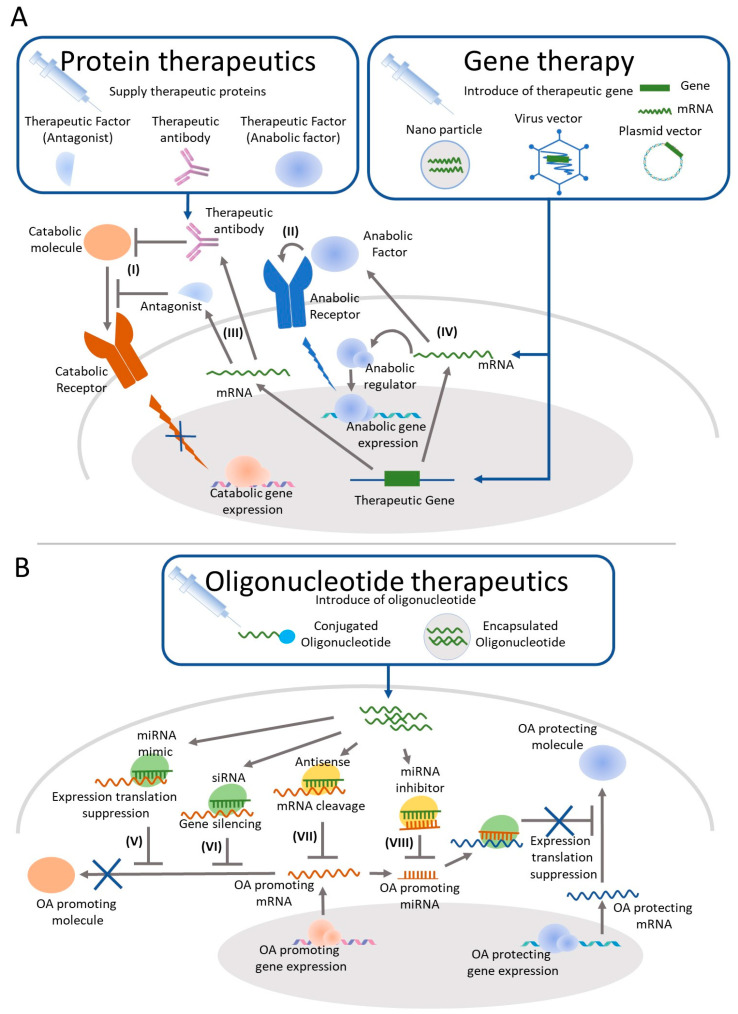
Current and potential targets of DMOADs for IA injection. (**A**) Current drug targets of protein therapeutics candidates for OA aim to block the interaction between proinflammatory cytokines and their receptors (I) or to supply cartilage protective proteins to the joint (II). The attempts of gene therapy candidates aim to supply proteins, which have anti-inflammatory (III) or cartilage repair effects (IV) instead of IA administration of recombinant protein. (**B**) OA progression may be controlled by reducing the protein production involved in pathological conditions through expression translation suppression by miRNA (V), gene silencing by siRNA (VI), or mRNA cleavage by antisense (VII). MicroRNA inhibitors inhibit OA promoting miRNAs (VIII) and may restore the expression of OA protecting proteins and may contribute to suppress OA progression.

The molecular mechanism of cartilage degeneration in OA and an understanding of target molecules that are the key to OA control are rapidly increasing. Once a target molecule is identified, it is possible to obtain DMOAD candidates by various drug discovery methods such as protein therapeutics, gene therapy, and nucleic-acid therapeutics (Figure 1). Each approach has pros and cons and appropriate modality for DMOADs differs depending on the expected mechanism of action and the target cells (Table 5).

Since protein therapeutics do not penetrate the cell membrane, this approach is applicable to target molecules such as cell surface receptors and secretory factors. Protein therapeutics can be expected to have high specificity for target molecules. Many drugs have been put into practical use and drug discovery procedures have been established. Although IA injection can deliver the protein therapeutics directly, even macromolecules such as proteins and hyaluronic acid are excreted from the joints in few hours or a day [120]. Technological advances such as modification of amino acid sequence [121], chemical modification [122], and development of sustained-release carrier [7,120,123] will contribute to the development of DMOADs. For oligonucleotide therapeutics, once the gene sequence of the target molecule is known, a method for quickly designing drug candidates has almost been established. Once the method of transfection into cells is established, the existing method can be used for oligonucleotide therapeutics for other targets [124]. Since oligonucleotide therapeutics are based on gene sequences, they are expected to act specifically on the target. They can be manufactured by chemical synthesis making it easy to set standards and control quality. Oligonucleotide therapeutics act as siRNA and can hybridize only to the target mRNA to reduce specific proteins’ synthesis. Oligonucleotide therapeutics aimed at supplementing or inhibiting miRNAs may act on multiple proteins. After being taken up into cells, their effect lasts for a certain period of time [110,125]. Oligonucleotide therapeutics can be inexpensively produced by chemical synthesis as distinct from biologically produced recombinant proteins and viral vectors. This is important for a candidate for the treatment of OA, which may be used for long periods in large numbers of patients. Using gene therapy, both intracellular and extracellular molecules can be expressed, either transiently or in response to a specific signal depending on the design of the vector [126]. There is also the possibility of the practical application of vectors that express siRNA [127,128,129], miRNA [130,131], and antisense RNA [132]. The ability to localize and supplement proteins through oligonucleotide therapeutics for long-term is considered to be one of the great advantages of gene therapy. However, so far, gene therapy has rarely been put to practical use in areas other than cancer and serious congenital diseases [133]. In those who have antibodies to viral vectors, there are concerns about reduced introduction efficiency and adverse events. In addition, because viral vectors or plasmid is biologically manufactured, it is difficult to control the quality standard, and the manufacturing cost is considered to be higher than that of oligonucleotide therapeutics. Gene therapy for OA may make significant progress if the use of gene therapy in high-severity diseases accumulates and safety concerns diminish.

There are several obstacles for development of DMOADs. Generally, surgically induced OA models have been used to evaluate in vivo efficacy. These models reflect some aspect of post traumatic OA in human, however heterogeneous nature of human OA should be considered. It has been reported that 12% of the overall prevalence of symptomatic OA is attributable to post-traumatic OA [134]. The pathophysiology of disease models used in preclinical research may be distinct from that of the majority of patients with OA. It should be considered to select appropriate experimental model, such as naturally occurring or genetically modified spontaneous models, surgically or chemically induced model or non-invasive models, which can replicate the targeted etiology in human OA [135]. For heterogeneity of human OA, the new classification for distinguishing different phenotype of OA is proposed [136]. It is expected that more precise OA classification will enable the selection of appropriate target patients for clinical trials according to the mechanism of action of the test drug [137]. Meanwhile establishment of reliable outcome measures, which can follow the course of disease and effect of intervention, are desired. The great potential of functional or compositional MRI for noninvasive assessment of tissue-structure changes in OA has been reported [138]. The selection of suitable experimental models, target patients, and appropriate outcome measure would be crucial for the successful development of new therapies.

## 8. Conclusions

OA progresses gradually over a long period of time and degeneration and damage to articular cartilage are irreversible. It is important to intervene in cartilage degeneration from an early stage and control its progression. Thus, IA injection of DMOAD at point-of-care may be one of the treatment options. In addition to conventional chemical compounds and protein therapeutics, DMOAD may be created from state-of-the-art gene therapy and oligonucleotide therapeutics.

## Figures and Tables

**Table 2:** Protein therapeutics candidates in OA animal models with reported efficacy.

Protein		OA Animal Models	References
GH	Growth hormone	Rat TMJ-MIA model	[44]
		Rabbit collagenase injection model	[45]
HB-IGF-1	Humanized insulin like growth factor-1 fusion protein with a heparin-binding domain for targeting to cartilage	Rat MMx model	[46]
FzD7 CRD	Recombinant-Frizzled 7-cysteine-rich domain designed to inhibit Wnt3a/β-catenin signaling	Mouse DMM model	[47]
rhMidkine	rhMidkine	Mouse DMM model	[48]
IL4-10 FP	A fusion protein, the biological activity of IL-4 and IL-10 are preserved	Canine groove model	[49,50]
Sclerostin		Mouse tibial compression OA injury model	[51]
Atsttrin	An engineered protein composed of three tumor necrosis factor receptor (TNFR)-binding fragments of progranulin (PGRN)	Rat noninvasive ACL rupture model mouse ACLT model	[52]
rhGDF5	rh growth differentiation factor-5	Rat MMx model	[53]
rhPRG4	rh lubricin	Yucatan minipigs DMM model	[54]
		Rat ACLT model	[55]
CRB0017	recombinant monoclonal antibodies directed against ADAMTS5	STR/ort	[56]

Efficacy reported in an experimental animal model by IA injection in last 10 years in PubMed. TMJ-MIA, monosodium iodoacetate injection to temporomandibular joint; rh, recombinant human; MMx, the medial meniscus was resected; DMM, destabilizing the medial meniscus surgery; ACLT, anterior cruciate ligament transection; STR/ort, spontaneous osteoarthritis mouse.

**Table 4:** Oligonucleotide therapeutics candidates ^1^ with in vivo efficacy via IA injection.

Mode of Action	Oligonucleotide	Target Gene(s)	Outcomes	References
miRNA inhibitor	miRNA inhibitors antisense oligonucleotide	*miR-141/200c*	Recover SIRT1/modify IL-6/STAT3 pathway/prevent OA in mouse DMM model	[102]
		*miR-203*	Recover Erα/decrease cartilage degradation in postmenopausal OA rats	[103]
		*miR-21-5p*	Recover FGF18/attenuate the severity of OA in the mouse DMM model	[104]
		*miR-34-5a*	Protect cartilage in the DMM and high-fat diet/DMM mice	[105]
		*miR-146b*	Recover α2-macroglobulin/ prevent OA in mouse DMM model	[106]
		*miR-181a-5p*	Attenuate cartilage destruction, hypertrophic, apoptotic/cell death, and type II collagen breakdown markers in mouse DMM model	[107]
		*miR-128a*	Recover ATG12/slow articular tissue destruction in rat ACLT model	[108]
		*miR-449a*	Recover SIRT 1/Prevent cartilage degradation in rat DMM model	[109]
mRNA inhibition	siRNA for target genes	*Hif-2α*	Prevent cartilage degeneration in ACLT/DMM mice	[110]
		*Mmp13*	Delay cartilage degradation in mouse DMM model	[111,112]
		*Yap*	Ameliorate OA development and reduce subchondral bone formation in ACLT mice	[113]
		*Thr*	Reduce angiogenic activities in subchondral bone ameliorated cartilage degradation in mouse DMM model	[114]
		*FoxC1*	Decrease β-catenin, ADAMTS-5, fibronectin, MMP3, and MMP13/decrease cartilage destruction in mouse DMM model	[115]
miRNA supplement	miR-210 mimic	Not mentioned	Upregulate *Col2a1* expression in the meniscus cells and VEGF and FGF2 expression in the synovial cell/enhance repair of the meniscus and prevent cartilage degeneration in rat DMM model	[116]
	miR-26a/26b mimic	*Fut4*	Promote chondrocytes proliferation and inhibit apoptosis/attenuate OA progression in rat ACLT-MMx model	[117]
	miR-145 mimic	*Mkk4*	Suppress the expression of MMP-3 and MMP-13, as well as p-MKK4, p-c-Jun, and p-ATF2/reduce cartilage destruction in rat MCLT-DMM model	[118]
	miR-140 mimic	Not mentioned	Reduce pathological scores and MMP-13 and ADAMTS-5 expression in rat ACLT-MMx model	[119]

^1^ Efficacy reported in an experimental animal model by IA injection. DMM, destabilizing the medial meniscus surgery; ACLT, anterior cruciate ligament transection; ACLT-MMx, ACL was transected, and the medial meniscus was completely resected; MCLT-DMM, medial collateral ligament transection and DMM.

**Table 5:** Summary of new approaches for disease-modifying osteoarthritis drugs (DMOADs).

	Protein Therapeutics	Gene Therapy	Oligonucleotide Therapeutics
Mechanism of action	Supply the required protein	Transduce target gene in cells Gene expression and translation is needed	Transfer to target cells Modulate the function or the fate of target mRNA
Application range of targets	For proteins that act extracellularly	Limitation in size of gene	Oligonucleotide sequence can be designed without off target effect
Delivery and distribution	Distribute to whole joint by IA	AAV vector provide cell type specific gene transfer	Drug delivery system for target need to be established
Retention time	Short Rapidly excreted from the joint	Vector: Continuous expression can be expected when transfected cells and vector retained mRNA: transient protein production	Effect will continue as long as oligonucleotide remain in cytosol
Control dosage and time	Possible	Amount of protein depend on transfection efficiency, host cell activity and etc. Promotor design provide regulated induction of protein	Possible
Relative manufacturing cost	High Biological manufacturing	High Biological manufacturing	Low Chemical synthesis”
Technical establishment and Safety concerns	Established Predictable	Approved mainly on life threatening disease Remain unknown risks?	Approved mainly in specific genetical disorder Remain unknown risks?

## Data Availability

No new data were created or analyzed in this study.
